# Successful use of HTF as a basal fertilization medium during SEcuRe mouse in vitro fertilization

**DOI:** 10.1186/s13104-023-06452-6

**Published:** 2023-08-24

**Authors:** Magdalena Wigger, Marco Schneider, Anni Feldmann, Sonja Assenmacher, Branko Zevnik, Simon E. Tröder

**Affiliations:** 1 Cluster of Excellence Cellular Stress Responses in Aging-Associated Diseases (CECAD), Faculty of Medicine and University Hospital Cologne, University of Cologne, Joseph-Stelzmann-Str. 26, 50931 Cologne, Germany; 2 In Vivo Research Facility, Faculty of Medicine and University Hospital Cologne, University of Cologne, Joseph-Stelzmann-Str. 26, 50931 Cologne, Germany

**Keywords:** In vitro fertilization, Sperm, Cryopreservation, Mouse models, Genetic engineering, Assisted reproductive technologies

## Abstract

**Objective:**

The ever-increasing number of genetically engineered mouse models highlights the need for efficient archiving and distribution of these lines. Sperm cryopreservation has become the preferred technique for the majority of these models due to its low requirement of costs, time, and experimental animals. Yet, current in vitro fertilization (IVF) protocols either exhibit decreased fertilization efficiency for the most popular C57BL/6 strain, as recently demonstrated by us, or require costly and difficult-to-prepare media, respectively. As a result, we previously developed SEcuRe, a modified IVF protocol with low costs and high fertilization efficiency. The popular basal fertilization medium, Cook’s^®^ proprietary “Research vitro fert” (RVF), used in this protocol has recently been discontinued. As a result, the application of the SEcuRe approach and other IVF protocols employing this medium has been severely limited.

**Results:**

Here we show that human tubal fluid (HTF), a popular and widely available medium with a known composition, can be used as a basal fertilization medium instead of RVF. Comparison of RVF and HTF during 58 independent SEcuRe IVFs with cryopreserved C57BL/6 sperm revealed equal fertilization and live birth rates. In addition, we demonstrate that HTF has a substantially extended shelf-life by utilizing commercial HTF that was six months past its expiration date, yet did not affect fertilization during IVF or subsequent embryo development. This finding not only increases the economic value of our modified method, but also validates it once more. Our results demonstrate that common, shelf-life extended HTF can be used in SEcuRe IVF in place of now-discontinued RVF medium and ensure the applicability of the method, which we since termed SEcuRe 2.0. Our modified SEcuRe 2.0 strategy will assist researchers to efficiently archive and distribute genetically engineered mouse models in a cost-effective, easily adaptable, and 3R-compliant manner with minimal animal use.

**Supplementary Information:**

The online version contains supplementary material available at 10.1186/s13104-023-06452-6.

## Introduction

**Fig. 1:**
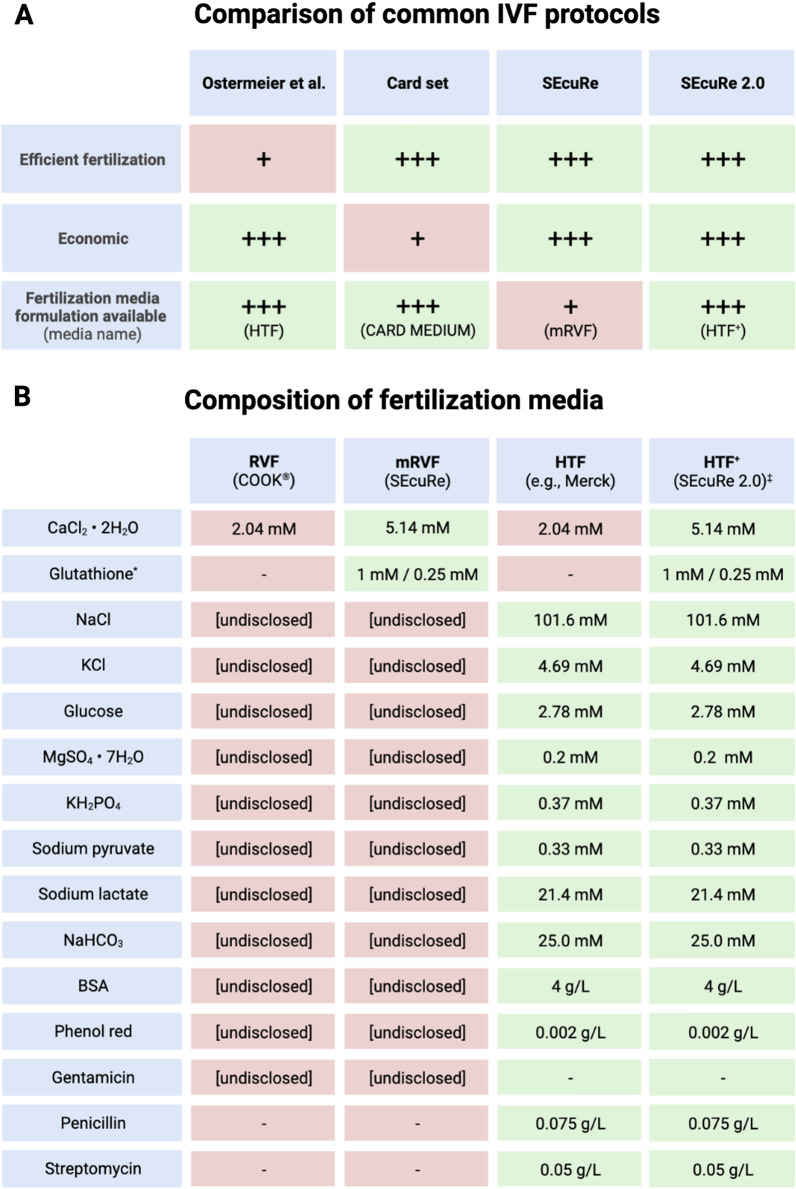
Comparison of SEcuRe 2.0 with published IVF protocols. Depicted are the relevant favorable (green) and unfavorable (red) characteristics (**A**) and the fertilization media composition (**B**) of SEcuRe 2.0 in contrast to published IVF protocols. ^*^Concentration dependent on sperm source (frozen/freshly harvested). ^‡^ The composition of HTF^+^ is identical to commercial CARD MEDIUM^®^ [4]

Genetically engineered mouse models have become the primary experimental tools in biomedical research over the past few decades. The development of highly adaptable genome-editing technologies, such as CRISPR/Cas9, has led to a rapid increase in the number of mutant mouse lines available [1]. Cryopreservation of sperm for future rederivation by IVF has become the preferred method for effective and economical archiving and distribution of these strains. In contrast to embryos, cryopreservation of sperm does not need breeding, requires fewer animals, and enables the production of many embryos upon rederivation [2]. There are currently two main protocols for sperm cryopreservation and IVF in use: one developed by Ostermeier et al. at The Jackson Laboratory (JAX) and the other developed by the Nakagata laboratory at the Center for Animal Resources and Development (CARD), often referred to as the CARD protocol (Fig. 1A) [3–7].

We have recently demonstrated that the CARD protocol results in significantly higher fertilization rates during IVF with cryopreserved sperm from the commonly used C57BL/6 and other strains compared to the Ostermeier et al. method [8]. Despite the availability of commercial media for the CARD protocol, budgetary restrictions continue to be a burden. For a cost-effective routine application of IVF, we have previously published our IVF protocol, which we termed SEcuRe (Simple Economical set-up for Rederivation), based on the chemistry of CARD with low costs in the range of the Ostermeier et al. method while retaining high fertilization efficiency [8]. To date, we have successfully applied this protocol to more than 300 individual IVFs in our laboratory, including one published study [9], achieving high fertilization efficiency with low expenses. However, one limitation of our SEcuRE protocol already discussed in the initial publication remained: it employs a proprietary medium, Cook’s^®^ RVF, as the foundation for the final fertilization medium, modified RVF (mRVF) (Fig. 1A, B). Fertilization medium is a crucial component of any IVF method. The Ostermeier et al. protocol uses HTF, a well-known mouse fertilization medium with published composition (Fig. 1B) [6, 7, 10]. Instead, the CARD protocol utilizes CARD MEDIUM^®^, which is published as HTF with an increased Ca^2+^ concentration and the addition of reduced glutathione (GSH) to improve fertilization [4, 11, 12]. By supplementing commercial RVF medium with Ca^2+^ and GSH, our SEcuRe protocol builds directly upon the CARD protocol. Due to the sudden discontinuation of the popular RVF medium by its manufacturer in early 2023, an urgent need exists for an alternative. HTF is a widely available and inexpensive fertilization medium that has been used successfully for decades in mouse IVF protocols [6, 10]. HTF is considered to be interchangeable with RVF medium during mouse IVF [3, 6, 13] but to our knowledge, this has never been evaluated side-by-side. Therefore, we sought to determine whether commercial HTF could replace proprietary RVF to establish an improved SEcuRe 2.0 protocol. With the original SEcuRe approach's benefits of high fertilization at low expenses and simplicity of use, we aimed to refine to a fully available media composition that is identical to the leading CARD protocol, without needing to rely on commercial sourced CARD MEDIUM^®^. (Fig. 1A). To increase the economic value of our method even more, we also wanted to show that HTF can be used well after its specified expiration date.

## Materials and methods

The protocol described in this article is published as an updated version of the original SEcuRe method on protocols.io (https://dx.doi.org/10.17504/protocols.io.261ge4j3yv47/v4) [8, 14].

### Mice

All animal protocols were performed in compliance with the European, national and institutional guidelines and approved by the State Office of North Rhine-Westphalia, Department of Nature, Environment and Consumer Protection (LANUV NRW, Germany; animal study protocol AZ 81-02.04.2019.A335). Mice were euthanized by cervical dislocation. All efforts were made to reduce animal suffering as much as possible. Animals were maintained in the CECAD Research Center, University of Cologne, Germany, in individually ventilated cages (Greenline GM500; Tecniplast) at 22 °C (± 2 °C) and a relative humidity of 55% (± 5%) under a 12-h light cycle on sterilized bedding (Aspen wood, Abedd, Germany) and with access to sterilized commercial pelleted diet (Ssniff Spezialdiäten GmbH) and acidified water ad libitum. The microbiological status was examined as recommended by Federation of European Laboratory Animal Science Associations (FELASA) and the mice were free of all listed agents including opportunists [15]. ARRIVE Guidelines 2.0 were followed during the preparation of the manuscript [16].

### IVF of mouse oocytes

All media were prepared according to the SEcuRe method [8, 14]. HTF^+^ fertilization medium for SEcuRe 2.0 was prepared on the day of the IVF by supplementing commercial EmbryoMax^®^ HTF (MR-070-D, Merck) with 1 mM reduced GSH (Sigma; G4251) and increasing the calcium concentration from default 2.04 mM to 5.14 mM with CaCl_2_ (Sigma; C7902) analogous as previously described for the mRVF fertilization medium preparation. EmbryoMax^®^ HTF was thawed upon arrival, divided into aliquots and stored at − 80 °C until use. Once thawed, the aliquots were stored at 4 °C for no longer than two weeks. On the day of the IVF, HTF^+^ was prepared from EmbryoMax^®^ HTF either before or 6 months after the expiration date of the manufacturer. Sperm donor mice were derived from several different mutant lines on a C57BL/6 genetic background as published previously [8]. Each IVF was performed with cryopreserved sperm pooled from two 10- to 20-week-old C57BL/6 mutant males of the same line. To increase comparability of the conditions tested for evaluation of the extended shelf-life of HTF, pooled sperm from two 10–14-week-old males from the same line on a pure C57BL/6N background was used for all conditions. Oocytes were harvested from two 3–4-week-old (i.e., 12–14 g body weight) C57BL/6NRj or C57BL/6JRj females, depending on the genetic background of the sperm donor, purchased from Janvier Labs as published [8, 14] and acclimatized for at least five days. Two randomly chosen females were used for each condition to ensure a sufficient number of oocytes and independent experiments were performed at least three times to allow statistical evaluation. Fertilization rates were calculated 24 h after IVF and expressed as a percentage of the total number of fertilized oocytes that reached the 2-cell stage. To exclude counting parthenogenetic embryos from potentially infertile males, two IVFs with fertilization rate below 20% were removed from the retrospective analysis of 60 experiments. Embryos were cultured in vitro in a CO_2_ incubator (5% CO_2_, atmospheric O_2_, 37 ℃, 95% humidity) in M16 for an additional 72 h, and the percentage of blastocysts developed from 2-cell stage embryos was documented if stated. Embryo transfer of 2-cell stage embryos into recipients was performed as described and live birth rates calculated by the number of pups born per embryos transferred to delivering recipients assessed one day after the expected delivery date [8].

### Statistical analysis

Prism 9 (GraphPad; version 9.51) was used to create graphs and to calculate statistical significance, as well as descriptive statistics. All data were examined for normality (Shapiro–Wilk test). Unpaired ordinary one-way ANOVA with Tukey’s post hoc test was used to evaluate the statistical significance of data displaying Gaussian distribution for ≥ three data sets (parametric). Additionally, unpaired Kruskal–Wallis with Dunn’s post hoc test and Brown-Forsythe and Welch ANOVA with Dunnett’s post hoc test were performed to support failure to reject the null hypothesis of equal means to show that the IVFs performed equally well under the tested conditions. Unpaired, two-tailed Mann–Whitney test was used for the comparison of two data sets representing non-Gaussian distribution (nonparametric). Unpaired, two-tailed Student’s t test, Welch’s t test, and Kolmogorov–Smirnov test were additionally performed to support failure to reject the null hypothesis of equal medians to show that the protocols perform equally well. Below a *p*-value of 0.05, differences in the results were considered significantly different. Group allocations were not blinded throughout the experiments and data analysis. No data or animals were excluded from the study except for the data of one repetition following the development of 2-cell-stage embryos after IVF to blastocysts, which were lost due to human error and two IVFs during the retrospective analysis due to < 20% fertilization rate. Cofounders were minimized by pooling of the males and random selection of the females. The primary data of the study are depicted in Additional file 1: C.

## Results and discussion

The manufacturer has discontinued the basal fertilization medium employed in our published SEcuRe protocol, urging the need for a replacement. We began by exchanging previously used commercial RVF medium by a widely used commercial HTF (EmbryoMax^®^, Merck). The final fertilization media was prepared by adding Ca^2+^ and GSH to either RVF or HTF, resulting in mRVF for conventional SEcuRe or in HTF^+^ for novel SEcuRe 2.0, respectively (Fig. 1AB). We named the latter fertilization medium HTF^+^ to reflect the addition of components to standard HTF. HTF^+^ is thus an inexpensive and simple-to-prepare fertilization medium with the same chemical composition as CARD MEDIUM^®^ [4]. We also attempted to increase the shelf-life of HTF, as it is common to discard expired media due to the small quantities required in IVFs compared to the large containers it is sold in. Therefore, we prepared HTF^+^ from commercial HTF medium that had been expired for at least six months (HTF 6 m expired) and used it in our SEcuRe 2.0 protocol.

**Fig. 2:**
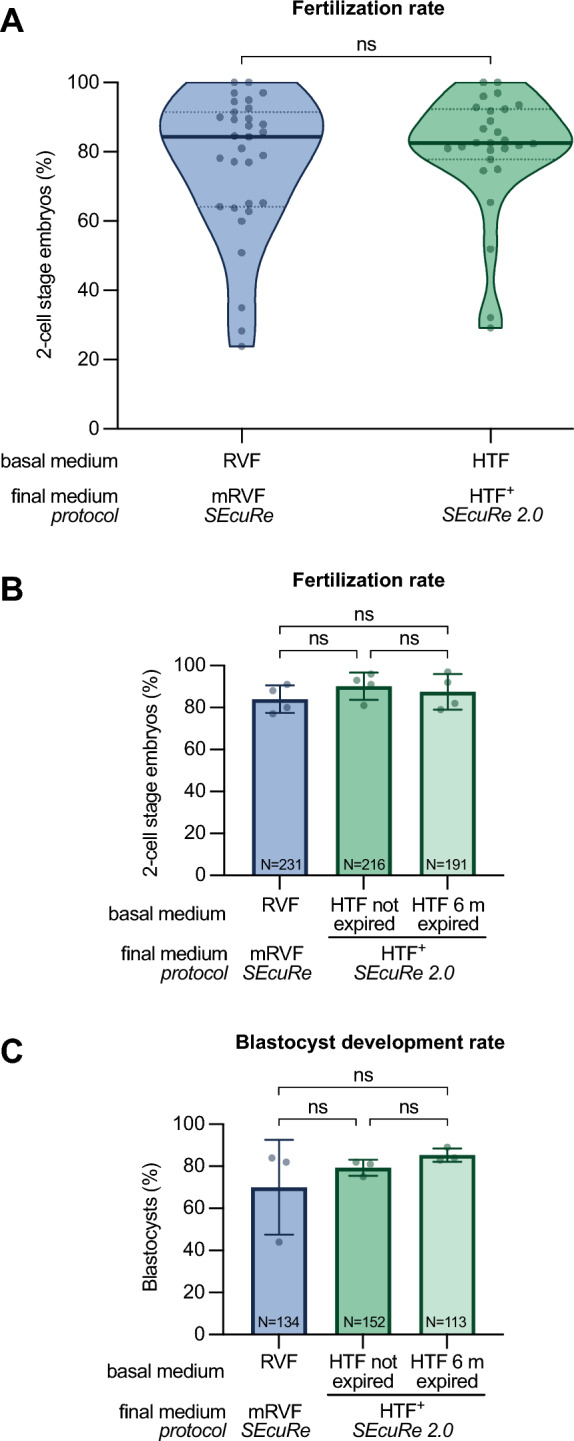
Validation of HTF as a basal fertilization medium in SEcuRe 2.0. **A** Comparison of the median fertilization rates using either RVF (SEcuRe) or HTF (SEcuRe 2.0) as the basal fertilization medium. Each dot represents an IVF performed with cryopreserved sperm from a single mutant C57BL/6 line. The fertilization medium as well as the basal fertilization medium are depicted. **B** Fertilization rates with shelf-life extended HTF compared to non-expired HTF or RVF as basal fertilization medium. The fertilization medium as well as the basal fertilization medium are depicted. IVF was performed using SEcuRe with cryopreserved sperm from C57BL/6N lines. Quantification of the fertilization rates are displayed from four independent experiments by assessing the percentage of 2-cell stage embryos 24 h later (n = 4). **C** Evaluation of the blastocyst developmental rate of the embryos from three independent experiments by quantification of 2-cell stage embryos reaching the blastocyst stage after 72 h of in vitro culture (n = 3). Thick lines in the violin plots indicate median fertilization rates and dotted lines the first and the third quartile. ns = non-significant (Mann–Whitney test). Dots in bar charts represent the fertilization rate from each experiment and bars represent means ± standard deviations from all experiment. N indicates the total number of oocytes (fertilization rate) or 2-cell stage embryos (blastocyst developmental rate) and ns = non-significant [ordinary one-way ANOVA (**A**) and Mann–Whitney test (**B**)]

To validate our approach, we retrospectively analyzed the in vitro fertilization rate of C57BL/6 oocytes with cryopreserved sperm from genetically engineered males with standard mRVF fertilization medium (SEcuRe) and novel HTF^+^ fertilization medium (SEcuRe 2.0). We considered the outcome of 31 independent experiments performed with standard mRVF and found that out of a total of 1406 inseminated oocytes 1065 developed to the 2-cell stage resulting in a median fertilization rate of 84.4% (Fig. 2A left violin plot). Strikingly, IVF with novel HTF^+^ led to the development of 1485 2-cell embryos out of 1998 inseminated oocytes achieving a virtually identical median fertilization rate of 82.5% in 27 independent experiments (Fig. 2A right violin plot). We conducted various statistical hypothesis tests, including the most appropriate Mann–Whitney test, to thoroughly examine the differences between our two conditions. All of these tests, which were chosen without bias, indicated the absence of a significant difference in the medians of the two conditions (Additional file 1: B). To confirm the developmental capacity of embryos generated using HTF^+^ in vivo*,* we compared the birth rates after embryo transfer of 2-cell embryos produced with either fertilization medium. As expected, we found no statistically significant difference between the mean birth rates using embryos produced with mRVF and HTF^+^ (28% ± 9% and 31% ± 6%, respectively; Additional file 1: A) These finding prove that HTF can be used instead of discontinued RVF without any reduction in fertilization efficiency and live birth rate.

We further aimed to increase the method’s economic value by preventing premature disposal of expired HTF prevalent in many IVF laboratories. To this end, we compared the IVF rate of oocytes with cryopreserved sperm from mice on a pure C57BL/6N background using either standard mRVF fertilization medium (SEcuRe) or HTF^+^ fertilization medium (SEcuRe 2.0) prepared from non-expired or 6 m expired HTF medium (Fig. 2B). Strikingly, we observed no significant difference in the mean fertilization efficiency in four independent experiments when six months expired HTF was used to prepare the HTF^+^ (87% ± 8.4%; Fig. 2B right column) in contrast to both, HTF^+^ prepared from non-expired HTF (90% ± 6.5%; Fig. 2B middle column) or mRVF (84% ± 6.6%; Fig. 2B left column). This reveals a substantially longer shelf-life for HTF and will eventually thereby further reduce the costs of IVF laboratories employing our SEcuRe 2.0 method. Following the development of a total of 399 embryos up to the blastocyst stage in three independent experiments, we found no significant differences in the mean developmental capacity of embryos fertilized in HTF^+^ from either expired HTF (85% ± 3.2%; Fig. 2C right column) or non-expired HTF (79% ± 3.8%; Fig. 2C middle column) or in mRVF (70% ± 22.5%; Fig. 2C left column). This further demonstrates the interchangeability of RVF and HTF and validates our SEcuRe 2.0 protocol once again. To support our findings, we once again employed various unbiasedly chosen statistical hypothesis tests, including the most appropriate one-way ANOVA with Tukey’s post hoc test, which demonstrated the absence of a significant difference in the means of fertilization and blastocyst developmental rates among the three conditions (Additional file 1: B). In conclusion, these findings show the viability of shelf-life extended, widely utilized HTF in SEcuRe 2.0 as a replacement for the discontinued RVF medium utilized in SEcuRe. We therefore changed the online version of our SEcuRe approach on protocols.io to SEcuRe 2.0 to reflect any modifications made to the initially published approach [8, 14].

The redundant function of RVF and HTF medium found in this study is consistent with published IVF protocols. For instance, Robert Taft (JAX) recommends either using RVF or HTF in its popular and very robust IVF protocol which has been used successfully with a wide range of different genetic backgrounds [5–7, 13]. In this context, our study provides for the first time necessary experimental evidence for the universal interchangeability of the popular but discontinued RVF and HTF for IVF protocols using this medium. In addition, the average fertilization rates and blastocyst developmental rates are comparable to those obtained in the initial publication of the SEcuRe method [8]. This validates the current findings once again and proves the reproducibility of our SEcuRe protocol. The results from over 30 independent IVF procedures with over 2000 embryos in this study furthermore proves the robustness of our novel SEcuRe 2.0 approach. In summary, we have demonstrated the applicability of shelf-life extended, widely used HTF as a replacement for the discontinued RVF medium providing researchers with our modified SEcuRe 2.0 protocol, a cost-effective and easily adaptable IVF method with the chemistry of the most popular CARD protocol for 3R favorable rederivation of mouse models from cryopreserved sperm.

## Limitations

We have yet to confirm the fertilization efficiency of SEcuRe 2.0 with genetic backgrounds other than the popular C57BL/6. We are confident that our SEcuRe 2.0 method is as robust as the widely used CARD protocol because HTF^+^ has the same composition as CARD MEDIUM^®^ [4]. Furthermore, to reduce animals used in research in accordance with the 3Rs, we decided to transfer only a modest number of embryos to recipients in vivo in the current study [17, 18]. However, we have previously observed no differences in the developmental capacity of embryos derived by different IVF protocols once fertilized. In addition, we chose to use only one commonly recommended commercial HTF [3, 6] in the current study but expect that any high-quality HTF designated for mouse IVF can be used in our SEcuRe 2.0 protocol due to its identical composition. HTF is offered by several companies, so the discontinuation of this medium and consequent inapplicability of our strategy are extremely unlikely in the future. Ultimately, it is also possible to prepare HTF in-house, although this is challenging, and we thus recommend the use of commercial HTF.

### Supplementary Information

**Additional file 1.** Additional statistical analysis and primary data.

## Data Availability

All data generated or analyzed during this study are included in this published article and its supplementary information files.

## Abbreviations

CARD: Center for Animal Resources and Development
GSH: Reduced glutathione
HTF: Human tubal fluid
IVF: In vitro fertilization
JAX: The Jackson Laboratory
m: Months
mRVF: Modified RVF
RVF: Research vitro fert
SEcuRe: Simple Economical set-up for Rederivation
