# Supplementary material for: Successful use of HTF as a basal fertilization medium during SEcuRe mouse in vitro fertilization
Source: BMC Res Notes. 2023 Aug 24;16:184. doi: 10.1186/s13104-023-06452-6 (PMC10463834; doi:10.1186/s13104-023-06452-6)
Supplement: Supplementary file 1 — Additional file 1. Additional statistical analysis and primary data. [file 13104_2023_6452_MOESM1_ESM.pdf]

Additional file 1

Successful use of HTF as a basal fertilization medium during SEcuRe mouse *in vitro* fertilization

Magdalena Wigger, Marco Schneider, Anni Feldmann, Sonja Assenmacher, Branko Zevnik, Simon E. Tröder

A

Live birth rates

mRVF (SEcuRe)			
**Experiment**	**No. of 2-cell embryos transferred[a]**	**No. of liveborn**	**Birth rate [%][b]**
1	88	10	11%
2	88	30	34%
3	88	25	28%
4	23	9	39%
5	88	26	30%
6	88	25	28%

Mean	28%
SD	9%

HTF+ (SEcuRe 2.0)			
**Experiment**	**No. of 2-cell embryos transferred[a]**	**No. of liveborn**	**Birth rate [%][b]**
1	44	16	36%
2	84	27	32%
3	66	15	23%
4	41	14	34%

Mean	31%
SD	6%

[a] number of 2-cell embryos transferred into delivering recipient mice.
[b] no statistically significant differences between live birth rates of mRVF vs. HTF$^+$ (t test).

B

Statistical hypothesis testing

p-values related to data shown in Fig 2A

	SEcuRe vs. SEcuRe 2.0
Mann-Whitney test	0.6122
Welch's t test	0.4809
Kolmogorov-Smirnov test	0.5387
Student's t test	0.4855

All tests are unpaired and two-tailed and considered significant below a *p*-value of 0.05

p-values related to data shown in Fig 2B

	RVF vs. HTF not expired	RVF vs. HTF 6 m expired	HTF not expired vs. HTF 6 m expired
Ordinary one-way ANOVA with Tukey's post hoc test	0.4698	0.7778	0.8548
Brown-Forsythe and Welch ANOVA with Dunnett's post hoc test	0.4957	0.8814	0.9351
Kruskal-Wallis with Dunn's post hoc test	0.4219	0.9070	>0.9999

All tests are unpaired and considered significant below a *p*-value of 0.05

p-values related to data shown in Fig 2C

	RVF vs. HTF not expired	RVF vs. HTF 6 m expired	HTF not expired vs. HTF 6 m expired
Ordinary one-way ANOVA with Tukey's post hoc test	0.6839	0.3944	0.8496
Brown-Forsythe and Welch ANOVA with Dunnett's post hoc test	0.8569	0.6447	0.2426
Kruskal-Wallis with Dunn's post hoc test	>0.999	0.4598	0.1520

All tests are unpaired and considered significant below a *p*-value of 0.05

C

Primary *in vitro* data

Related to Fig 2A

SEcuRe			SEcuRe 2.0		
ID	No. of oocytes	No. of 2-cell embryos	ID	No. of oocytes	No. of 2-cell embryos
1	38	30	1	50	26
2	42	34	2	23	19
3	30	27	3	33	32
4	65	39	4	46	43
5	46	30	5	45	35
6	20	7	6	41	33
7	51	26	7	85	70
8	98	63	8	199	64
9	56	50	9	85	78
10	70	64	10	54	48
11	70	44	11	34	34
12	32	27	12	48	40
13	47	30	13	39	36
14	58	51	14	27	22
15	36	34	15	200	150
16	52	40	16	134	100
17	33	32	17	139	91
18	46	13	18	17	17
19	42	10	19	79	23
20	26	22	20	63	51
21	56	48	21	226	183
22	32	28	22	50	48
23	55	43	23	56	52
24	29	29	24	60	52
25	34	33	25	70	60
26	26	26	26	40	33
27	48	37	27	55	45
28	26	17			
29	54	50			
30	48	43			
31	40	38			

C (continued)

Related to Fig 2BC

Experiment number	Basal fertilization media	Age of sperm donor	No. of oocytes	No. of 2-cell embryos	Fertilization rate	No. of blastocyst	Blastocyst formation rate
1	RVF	11 weeks	69	61	88%	51	84%
	HTF not expired		69	63	91%	51	81%
	HTF 6 m expired		39	38	97%	34	89%
2	RVF	14 weeks	69	63	91%	no data	
	HTF not expired		46	43	93%		
	HTF 6 m expired		59	54	92%		
3	RVF	11 weeks	44	34	77%	15	44%
	HTF not expired		47	45	96%	37	82%
	HTF 6 m expired		38	30	79%	25	83%
4	RVF	12 weeks	49	39	80%	32	82%
	HTF not expired		54	44	81%	33	75%
	HTF 6 m expired		55	45	82%	38	84%
